# Recurrent borderline phyllodes tumor of the breast submitted to mastectomy and immediate reconstruction: Case report

**DOI:** 10.1016/j.ijscr.2019.05.032

**Published:** 2019-06-05

**Authors:** Bruno Bisognin Garlet, Luciano Zogbi, Juliana Piveta de Lima, Paulo Pereira de Souza Favalli, Frederico Diefenthaeler Krahe

**Affiliations:** a Department of Surgery, Federal University of Rio Grande (FURG), Visconde de Paranaguá Street, No. 102, 96203-900, Rio Grande, Brazil; b Postgraduate Program in Nursing, Federal University of Rio Grande (PPGEnf - FURG), Visconde de Paranaguá Street, No. 102, 96203-900, Rio Grande, Brazil; c Department of Plastic Surgery, Moinhos de Vento Hospital, Ramiro Barcelos Street, No. 910/604, 90035-004, Porto Alegre, Brazil; d Department of Breast Surgery, Moinhos de Vento Hospital, Ramiro Barcelos Street, No. 910/604, 90035-004, Porto Alegre, Brazil

**Keywords:** Phyllodes tumor, Breast, Mastectomy, Mammaplasty, Plastic surgery, Case report

## Abstract

**Introduction:**

Phyllodes tumors are biphasic fibroepithelial tumors that account for fewer than 1% of mammary tumors. They usually appear in middle-aged women, with an average size of 4–7 cm and rapid growth.

**Presentation of case:**

A 25-year-old woman sought care after excision of recurrent nodules in the right breast, with a diagnosis of borderline phyllodes tumor. She had no new lesions on physical examination. Imaging studies revealed an anechoic collection and nodular areas in a previous surgical site, correlated with pathology. Immuno-histochemical examination was positive for vimentin, calponin, Ki-67 and estrogen receptor. Systemic staging did not show metastases. Skin-reducing mastectomy was performed on the right side, followed by reconstruction and left reduction mammaplasty for symmetry. At 8-month follow-up, there was no relapse of the lesions on clinical and ultrasonographic examinations.

**Discussion:**

Phyllodes tumors are rare neoplasms characterized by rapid growth, occurring in women between 35–55 years of age. They are classified as benign, borderline and malignant, according to histological parameters. There are reports of genetic mutations in TP53 associated with malignant phyllodes tumor. On immunohistochemistry, there is a greater tendency to malignancy in tumors with Ki-67 and estrogen receptor expression. Imaging methods may aid in diagnosis, which is only definitive after excision and histopathological analysis of the tumor. These tumors have high local recurrence rates and possibility of metastases, closely related to histology.

**Conclusion:**

Phyllodes tumors are a great challenge to the surgeon. Treatment is surgical, with wide excision; radiotherapy and chemotherapy are controversial.

## Highlights

• Phyllodes tumors of the breast are rare fibroepithelial neoplasms.
• They are histologically classified into benign, borderline and malignant variants.
• Anatomopathological examination is considered the definitive diagnostic method.
• They have a high rate of local recurrence and the possibility of metastases.
• Surgery is the definitive treatment and adjuvant therapy is controversial.

## 1 Introduction

Phyllodes tumors (PTs) account for fewer than 1% of mammary tumors [1]. Its name derives from the Greek *phyllon* (leaf) because of its lobed histological appearance. It is also known as cystosarcoma phyllodes, adenomatous myxoma and pseudosarcoma adenoma [[1], [2], [3]]. PTs are common in middle-aged women, with an average size of 4–7 cm and rapid growth [1,3,4]. They are fibroepithelial tumors, composed of stromal and epithelial elements, differing from fibroadenomas (FA) by the greater abundance of the stromal component [3]. Because of local recurrence and metastatic potential, they are surgically treated with wide local excision and mastectomy [[2], [3], [4], [5]].

The objective of this study, reported according to SCARE criteria [6], is to describe a case of recurrent borderline PT in a young patient undergoing mastectomy and immediate breast reconstruction in a private hospital.

## 2 Presentation of case

A Caucasian female sought care at 22 years of age for evaluation of a mass characterized by progressive growth located laterally in the right breast, measuring 3.3 cm; she underwent surgical excision, with anatomic-pathological examination suggestive of FA. One year later, the patient had a palpable mass of 4.7 cm in the same location, and a lumpectomy was performed, with a repeated diagnosis of FA. At eight and 12 months thereafter, two new tumors, both 4.5 cm, were resected, and benign PTs were diagnosed with compromised resection margins. After another eight months, at the age of 25, three new lesions appeared; all nodules were excised, with borderline PT diagnosis, positive margins for the largest (3 cm) and benign PT for the other two.

**Fig. 1:**
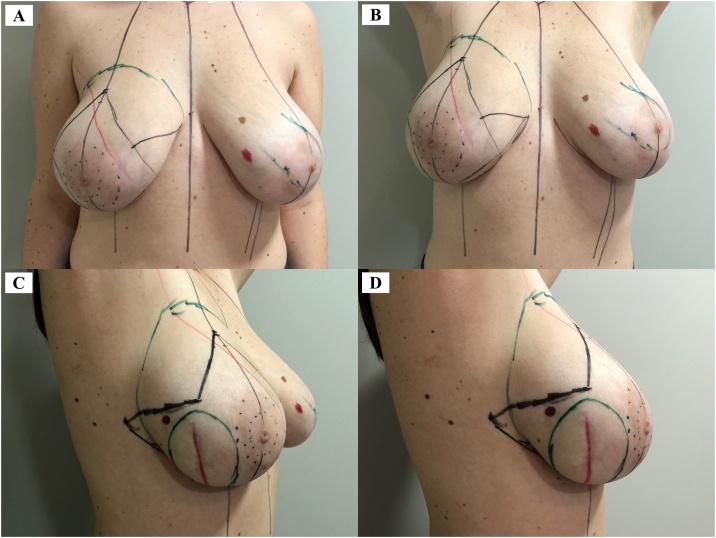
Preoperative demarcation lines: incision line (black), breast delimitation and pericicatricial tissue (green) and previous scar (red). A and B. Front view. C and D. Right lateral view.

Over time, a 6-cm scarring lesion was presented, laterally in the right breast, consequent to the previous procedures, and thickening of the underlying tissue, without phlogosis (Fig. 1). On palpation, no new nodules or axillary lymph nodes were identified. The immuno-histochemical examination showed positivity for vimentin, calponin, Ki-67 and estrogen receptor in the epithelial component. Mammography, ultrasonography (US) and magnetic resonance imaging (MRI) showed an anechoic collection measuring 3.0 × 1.0 cm in a surgical site of the right breast, with persistent and slightly irregular late enhancement in the inferolateral margin of the collection, and an area between 0.4–0.7 cm, correlated with the previous pathology. Thoracic CT and abdominal US showed no metastases. Genetic testing revealed uncertain significance variants in BRCA1 and BRCA2, without mutation in PT53.

**Fig. 2:**
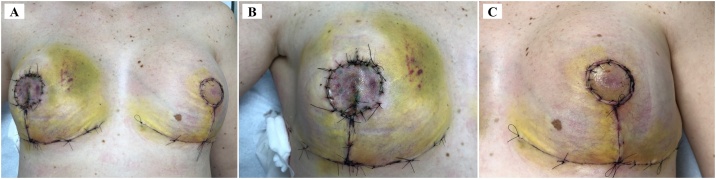
7th postoperative day. A. Front view. B. Product of right-sided skin-reducing mastectomy, followed by reconstruction with nipple-areola complex graft. C. After left reduction mammaplasty for symmetry with flap rotation of nipple-areola complex.

**Fig. 3:**
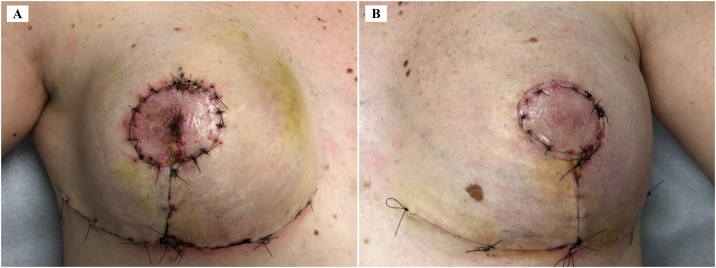
16th postoperative day. A. Right breast. B. Left breast.

**Fig. 4:**
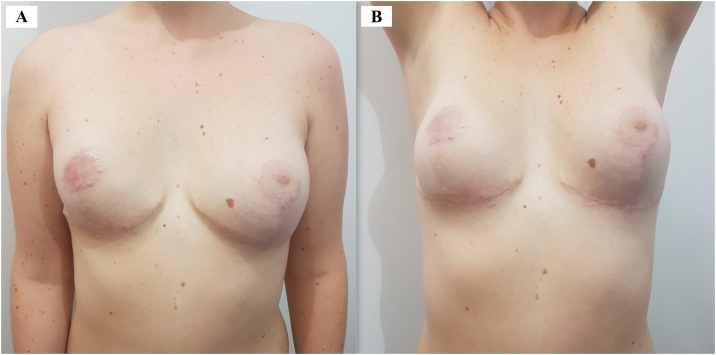
A and B. At 6-month postoperative follow-up.

All lesions occurred in the right breast and were asymptomatic. Because of the history of frequent relapses, a right-sided skin-reducing mastectomy was chosen, followed by reconstruction. The intraoperative frozen section showed a scarring lesion underlying the surgical marking and another nodular hemorrhagic lesion in a super-medial quadrant with resection margins greater than 1.0 cm in relation to the adjacent tissues. On the left, reduction mammaplasty was chosen for symmetry, because of the patient's large breast volume (Fig. 2, Fig. 3). The pathology showed no residual PT. At 8-month follow-up, there was no relapse of the lesions on clinical examination or US (Fig. 4).

## 3 Discussion

PTs are rare breast neoplasms characterized by rapid growth [1,7,8]. They usually appear in women between 35 and 55 years of age, with an average presentation at 45 years, occurring ten to 20 years after FA peak incidence, by contrast with this case, in which the patient manifested the disease before 25 years of age [[9], [10], [11], [12]]. On physical examination, PTs are usually rigid, well delimited, mobile, non-adherent to the skin, with an average size of 4–7 cm, as described previously [2,3,13]. They appear commonly in isolation; therefore, this case introduced diagnostic difficulty, because the patient presented with multiple nodules of various sizes [4].

Histologically, PTs have leaf-like cellular lobulations and biphasic fibro-epithelial constitution, with predominance of stromal elements over epithelial cells. The World Health Organization classifies them as benign (60–75%), borderline (13–26%) and malignant (10–20%) [2,3,5]. Because they do not present typical clinical manifestations, it is difficult to distinguish them macroscopically from FA and even to distinguish among their subtypes. The definitive diagnosis is histopathological, after complete excision of the tumor [3]. Characteristically, benign PTs show minimal cellular atypia, mitotic index ≤4/10 HPF and slightly increased stromal cellularity, the presence of which distinguishes them from FAs; borderline PTs show greater atypia, mitotic index between 5–9/10 HPF and moderate stromal cellularity, with possible areas of hemorrhage and necrosis; malignant PTs, which may be confused with mammary sarcoma or metaplastic spindle cell carcinoma, show marked atypia and stromal hypercellularity, mitotic count ≥10/10 HPF, infiltrative-expansive growth and presence of large necrohemorrhagic areas [2,8,13]. In our patient, the histopathological review of the last procedure performed before the mastectomy showed a borderline PT of 3.0 cm, with moderate cellular atypia, 9 mitoses/10 HPF and positive margins, and two other lower borderline PTs, also with moderate atypia, and mitotic indexes of 3/10 HPF and 5/10 HPF, respectively, without compromising the surgical margins. As for immunohistochemistry, there is a greater tendency towards malignancy in the context of Ki-67 positivity, in addition to positivity of estrogen receptors in the epithelial component, as in this patient [8,14].

Unlike breast cancer, which has a well-established association with mutations in the BRCA1 and BRCA2 genes, there remains insufficient evidence linking genetic variations to the development of PT. There are reports of mutations associated with malignant PT, as in the PT53 gene; this was absent in our patient, whose test showed only variants of uncertain significance in BRCA1 and BRCA2 [[14], [15], [16]].

Diagnostic imaging methods have low accuracy [13]. US and mammography generally show large, round, lobulated, well-delimited masses with a higher density than adjacent tissue, or may present a clear halo around the tumor because of rapid growth, without clearly distinguishing between the three subtypes of PT or even FA. Cystic areas, heterogeneous patterns without micro-calcifications, and acoustic enhancement on US are more suggestive of PT than FA, especially when associated with rapid growth and large volume. Volume >3 cm on mammography and cystic areas on US are suggestive of malignancy [1,3,4,17,18]. Solid portions with greater intensification within cystic spaces hemorrhagic on MRI suggest PT [18].

While malignant PTs have high potential for local recurrence and metastases, benign PTs tend to recur only locally [8]. Local recurrence rates are 3.6–8% for benign PT, 14% for borderline and 30–42% for malignant [1,4,13]. Although our patient presented with benign and borderline PTs, she had several recurrences, probably associated with compromised surgical margins, considered the main risk factor for recurrence [9,13,17,19]. Metastatic potential is also closely related to malignancy, with greater hematogenous spread, mainly pulmonary and bone, justifying systemic staging. The metastases rates are <1% for benign PTs, 1.6% for borderline and 16–22% for malignant [2,4,8]. The mean survival over ten years is 86%, ranging from 96.4% for benign PTs to 57.7% for malignant [13].

Treatment of PT is surgical, regardless of subtype, ranging from wide local excision to mastectomy. Currently, there is a greater tendency towards conservative approaches, because PT is rarely multifocal. Recent guidelines recommend that PT > 3 cm be resected with free margins ≥1 cm, with no need for axillary staging; lumpectomy may be indicated for small tumors, because it allows preservation and mammary function [3,4,17,20]. Some studies suggest that patients with benign and borderline PTs >5–10 cm or with other risk factors for recurrence and metastasis, malignant PTs, more than three recurrences or positive resection margin, or age above 50 years, should be subjected to mastectomy, preferably followed by immediate reconstruction [3,9,18]. Because our patient had progressive, borderline and more than three recurrent tumors, skin-reducing mastectomy on the right was chosen, removing all tumor mass, adjacent fibrotic tissue, and cutaneous scar tissue resulting from previous procedures, providing free and wide margins, as recommended [3,4,20]. For the immediate reconstruction, after analysis of the breast characteristics and the anthropometric relations of the patient, we decided to perform retro-muscular mammary implant, with inferior dermal flap, sutured to the edge of the pectoralis major muscle, and careful preparation of the right nipple-areola complex graft, to achieve the best aesthetic result. On the left side, mammaplasty was performed to achieve symmetry, followed by sub-glandular prosthesis implantation, with substantial volume reduction and flap rotation involving the nipple-areola complex, aiming at aesthetic and functional preservation. In patients with large breast volume and superficial cutaneous involvement, such as in our patient, this approach is associated with less aesthetic and psychological damage, enabling preservation of the nipple-areola complex and greater surgical radicality [5].

Adjuvant therapy has no proven efficacy in terms of disease-free survival, and was not provided in this case. Some studies, although controversial, suggest chemotherapy for metastatic PTs and radiotherapy for malignant PTs with positive margins, especially when local control is desired and surgical treatment is not possible. Furthermore, radiotherapy may be useful in recurrent PTs, because it appears to be associated with lower rates of local recurrence [7,8,17,20]. Because of the high risk of recurrence, postoperative clinical-radiological surveillance is recommended.

## 4 Conclusion

PT of breast is a rare neoplasm, posing great challenges to the surgeon. It presents high rates of local recurrence and possibility of metastases. Histopathological analysis is considered the definitive diagnostic method. Treatment is surgical, with wide excision, whereas radiotherapy and chemotherapy are controversial.

## Conflicts of interest

None to declare.

## Sources of funding

This research did not receive any specific grant from funding agencies in the public, commercial, or not-for-profit sectors.

## Ethical approval

Not applicable. Since this is a case report, not a research study, ethical approval has been exempt/not required.

## Consent

Written informed consent was obtained from the patient for publication of this case report and accompanying images. A copy of the written consent is available for review by the Editor-in-Chief of this journal on request.

## Author contribution

**Bruno Bisognin Garlet**: Conceptualization, Methodology, Investigation, Writing – Original Draft, Writing – Review & Editing, Visualization, Supervision, Project Administration.

**Luciano Zogbi**: Conceptualization, Methodology, Writing – Review & Editing, Visualization.

**Juliana Piveta de Lima**: Writing – Review & Editing, Visualization.

**Paulo Pereira de Souza Favalli**: Resources.

**Frederico Diefenthaeler Krahe**: Resources.

## Registration of research studies

Not applicable.

## Guarantor

Bruno Bisognin Garlet.

## Provenance and peer review

Not commissioned, externally peer-reviewed.

## References

[1] Stoffel E., Becker A.S., Wurnig M.C., Marcon M., Ghafoor S., Berger N. (2018). Distinction between phyllodes tumor and fibroadenoma in breast ultrasound using deep learning image analysis. Eur. J. Radiol. Open.

[2] Lakhani S.R., Ellis I.O., Schnitt S.J., Tan P.H., van de Vijver M.J. (2012). WHO Classification of Tumours of the Breast.

[3] Wang Y., Zhang Y., Chen G., Liu F., Liu C., Xu T. (2018). Huge borderline phyllodes breast tumor with repeated recurrences and progression toward more malignant phenotype: a case report and literature review. Onco Target. Ther..

[4] Wolbert T., Leigh E.C.N., Barry R., Traylor J.R., Legenza M. (2018). Early stage malignant phyllodes tumor case report. Int. J. Surg. Case Rep..

[5] Ciancio F., Innocenti A., Cagiano L., Portincasa A., Parisi D. (2017). Skin-reducing mastectomy and direct-to-implant reconstruction in giant phyllodes tumour of breast: case report. Int. J. Surg. Case Rep..

[6] Agha R.A., Borrelli M.R., Farwana R., Koshy K., Fowler A., Orgill D.P., For the SCARE Group (2018). The SCARE 2018 statement: updating consensus surgical CAse REport (SCARE) guidelines. Int. J. Surg..

[7] Wang Q., Su J., Lei Y. (2017). Recurrent malignant phyllodes tumor of the breast: a case report. Medicine.

[8] Tan B.Y., Acs G., Apple S.K., Badve S., Bleiweiss I.J., Brogi E. (2016). Phyllodes tumours of the breast: a consensus review. Histopathology.

[9] Abdul Hamid S., Rahmat K., Ramli M.T., Fadzli F., Jamaris S., See M.H. (2018). Radiopathological characteristics and outcomes of phyllodes tumor of the breast in Malaysian women. Medicine.

[10] Bumpers H.L., Tadros T., Gabram-Mendola S., Rizzo M., Martin M., Zaremba N. (2015). Phyllodes tumors in African American women. Am. J. Surg..

[11] Abusalem O.S., Al-Masri A. (2011). Phyllodes tumors of the breast. Mater. Sociomed..

[12] Parker S.J., Harries S.A. (2001). Phyllodes tumours. Postgrad. Med. J..

[13] Zhou Z.R., Wang C.C., Sun X.J., Yang Z.Z., Chen X.X., Shao Z.M. (2018). Prognostic factors in breast phyllodes tumors: a nomogram based on a retrospective cohort study of 404 patients. Cancer Med..

[14] Wang Y., Zhu J., Gou J., Xiong J., Yang X. (2017). Phyllodes tumors of the breast in 2 sisters: case report and review of literature. Medicine.

[15] Huszno J., Kołosza Z., Grzybowska E. (2019). BRCA1 mutation in breast cancer patients: analysis of prognostic factors and survival. Oncol. Lett..

[16] Liu I., Ide Y., Inuzuka M., Tazawa S., Kanada Y., Matsunaga Y. (2019). BRCA1/BRCA2 mutations in Japanese women with ductal carcinoma in situ. Mol. Genet. Genomic Med..

[17] Shah-Patel L.R. (2017). Malignant phyllodes breast tumor. Radiol. Case Rep..

[18] Rajesh A., Farooq M. (2017). Resection and reconstruction following recurrent malignant phyllodes–case report and review of literature. Ann. Med. Surg..

[19] Ren J., Jin L., Leng B., Hu R., Jiang G. (2018). Surgical excision and oncoplastic breast surgery in 32 patients with benign phyllodes tumors. World J. Surg. Oncol..

[20] Warner W.A., Sookdeo V.D., Fortuné M., Akhilesh M., Rao Adidam Venkata C., Mohammed W. (2017). Clinicopathology and treatment of a giant malignant phyllodes tumor of the breast: a case report and literature review. Int. J. Surg. Case Rep..

